# The loss of microglia activities facilitates glaucoma progression in association with *CYP1B1* gene mutation (p.Gly61Glu)

**DOI:** 10.1371/journal.pone.0241902

**Published:** 2020-11-10

**Authors:** Amani Alghamdi, Wadha Aldossary, Sarah Albahkali, Batoul Alotaibi, Bahauddeen M. Alrfaei

**Affiliations:** 1 Biochemistry Department, King Saud University (KSU), Riyadh, Saudi Arabia; 2 Stem Cells and Regenerative Medicine, King Abdullah International Medical Research Center (KAIMRC), Riyadh, Saudi Arabia; 3 King Saud Bin Abdulaziz University for Health Sciences (KSAU-HS), Riyadh, Saudi Arabia; The Second Affilated Hospital, Zhejiang University School of Medicine, CHINA

## Abstract

**Background:**

Glaucoma represents the second main cause of irreversible loss of eyesight worldwide. Progression of the disease is due to changes around the optic nerve, eye structure and optic nerve environment. Focusing on primary congenital glaucoma, which is not completely understood, we report an evaluation of an untested mutation (c.182G>A, p.Gly61Glu) within the *CYP1B1* gene in the context of microglia, astrocytes and mesenchymal stem cells. We investigated the behaviours of these cells, which are needed to maintain eye homeostasis, in response to the *CYP1B1* mutation.

**Methods and results:**

CRISPR technology was used to edit normal *CYP1B1* genes within normal astrocytes, microglia and stem cells *in vitro*. Increased metabolic activities were found in microglia and astrocytes 24 hours after *CYP1B1* manipulation. However, these activities dropped by 40% after 72 hrs. In addition, the nicotinamide adenine dinucleotide phosphate (NADP)/NADPH reducing equivalent process decreased by 50% on average after 72 hrs of manipulation. The cytokines measured in mutated microglia showed progressive activation leading to apoptosis, which was confirmed with annexin-V. The cytokines evaluated in mutant astrocytes were abnormal in comparison to those in the control.

**Conclusions:**

The results suggest a progressive inflammation that was induced by mutations (p.Gly61Glu) on *CYP1B1*. Furthermore, the mutations enhanced the microglia’s loss of activity. We are the first to show the direct impact of the mutation on microglia. This progressive inflammation might be responsible for primary congenital glaucoma complications, which could be avoided via an anti-inflammatory regimen. This finding also reveals that progressive inflammation affects recovery failure after surgeries to relieve glaucoma. Moreover, microglia are important for the survival of ganglion cells, along with the clearing of pathogens and inflammation. The reduction of their activities may jeopardise homeostasis within the optic nerve environment and complicate the protection of optic nerve components (such as retinal ganglion and glial cells).

## Introduction

Glaucoma is a major contributor to blindness. It predicted to affect 80 million people in 2020 and is the second major cause of irreversible loss of eyesight worldwide [1, 2]. It is a neurodegenerative disease consisting of a non-uniform group of ocular disorders characterised by a number of clinical features that include visual field defects, retinal ganglion cell death and progressive degeneration of the optic nerve [3, 4]. The international classification of childhood glaucoma by the Childhood Glaucoma Research Network (CGRN) and World Glaucoma Association (WGA) of childhood glaucoma divides the disease into two types: primary and secondary childhood glaucoma. Primary childhood glaucoma is further classified into primary congenital glaucoma (PCG) and juvenile open-angle glaucoma (JOAG) [5]. In children, the most common form of the disease is PCG (OMIM 231300), and is usually inherited as an autosomal recessive disease with incomplete penetrance [6]. The prevalence of PCG varies geographically from a rate of 1:10,000 in Western countries to 1:1,250 in the Romany population of Slovakia [7].

The cytochrome P450 family 1 subfamily B member 1 (*CYP1B1*; NM_000104.3) gene is the most commonly mutated gene in PCG in the Middle East [8], including Saudi Arabia [9] which has been validated in the western region of Saudi Arabia [10]. The *CYP1B1* enzyme has been found to be functional in various tissues, such as the ovaries, breast, prostate, colon, brain, and eye [11–13]. Defects in *CYP1B1* have been associated with immature eye development causing developmental anomalies, such as trabecular meshwork dysgenesis [14]. Although no data is available for other parts of Saudi Arabia, we suspect that *CYP1B1* mutation is still the most common mutation linked to PCG in Saudi Arabia based on the available studies [14, 15]. The PCG variant p.Gly61Glu in *CYP1B1* has been identified as the major disease-associated mutation in Saudi Arabia, representing 63% of all cases [14, 16]. It is not well understood how the *CYP1B1* mutation p.Gly61Glu damages the optic nerve or leads to blindness during disease progression. The optic nerve is supported by glial cells, or the neuroglia, which play a vital role in its maintenance. These cells occupy half of the brain space and are considered non-neuronal cells in the CNS. They are classified into three major cell types: astrocytes, oligodendrocytes and microglia [17]. Astrocytes are known to promote neuronal survival, myelination and synaptogenesis. In addition, they regulate neurotransmitters, ion exchange and the blood-brain barrier [18, 19]. Microglia are known to eliminate microbes, dead cells, cellular debris, excess synapses, protein aggregates and other particulates [20]. The molecular aspects and consequences of having the *CYP1B1* p.Gly61Glu variant in optic-nerve supporting cells are not understood. In this study, however, we showed the effects of the *CYP1B1* mutation p.Gly61Glu (G61E) on the supporting cellular components of the optic nerve, such as glial cells. We focus on microglia and astrocytes (which are parts of the glial cell population) and their responses to *CYP1B1* mutation.

## Materials and methods

### Primary cell extraction from animals

Healthy two-day-old male Sprague-Dawley rats weighing 5–6g were obtained from the King Saud University (KSU) animal facility in the College of Pharmacy. All animal involvements were ethically approved by King Abdullah International Medical Research Center Review Board, RC17/038/R. The isolation of astrocytes and microglia from the brains of these animals was performed using Hong Lian’s protocol [21]. The rats were decapitated, and their heads were placed in a 5% foetal bovine serum (FBS) (Catalogue #10099141, Gibco, United States of America [USA]), 1% Pen-Strep antibiotic (Catalogue #15140–122, Gibco, USA) and DMEM (Catalogue #11885–084, Gibco, USA) medium. The protocol was carried out in a sterile class II biosafety cabinet. The meninges were removed using forceps, and the cortices and hippocampi were collected. The tissues were homogenised and divided into two prepared tubes that contained trypsin, DNase, phosphate buffer saline (PBS), 10% FBS, 1% GlutaMAX (Catalogue #35050–061, Gibco, USA), 1% Pen-Strep antibiotic, DMEM, trypsin (Catalogue #12604–013, Gibco, USA), DNase (Catalogue #LS002007, Worthington Biochemical Corporation, USA) and PBS (Catalogue #10010–031, Gibco, USA). These tubes were vortexed and then incubated with 5% CO_2_ at 37°C for 20 mins (mixed carefully every 5 mins). 100μm and 40μm cell strainers were used, and centrifugation was performed at 400 × g for 5 mins. Each tube was washed twice with PBS and RBC lysis buffer. Pellets were taken and washed with 0.9M of sucrose. Again, centrifugation was performed at 600 × g for 5 mins to discard the supernatant. The pellets were pelleted on culture flasks containing 10% FBS, 1% GlutaMAX, 1% Pen-Strep and advanced DMEM. The flasks were vigorously tapped for a few days into the culture, which typically releases microglia from the culture flask while leaving astrocytes attached. Finally, the medium containing the microglia was placed into new flasks along with GM-CSF (Catalogue #PHC2013, Thermo Fisher Scientific, USA) growth factors. Additionally, new media with 10% FBS were added to the old flasks containing astrocytes. Proper ethical approvals were obtained for this project from KSU and the King Abdullah International Medical Research Center (KAIMRC) in Saudi Arabia. The purity of the microglia was 91%, whereas the purity of the astrocytes was 93.9% (see S2 Fig).

### CRISPR editing of target cells

A custom-designed CRISPR kit (Origene, Rockville, MD, USA) for *CYP1B1* (c.182G>A, p.Gly61Glu, a CRISPR pCas-Guide) was used, as previously described in other studies [22, 23]. In the pCas-Guide, the following modified sequence was used:

CGCGCCCCCGGGCCCGTTTGCGTGGCCACTGATCG[A]AAACGCGGCGGCG GTGGGCCAGGCGGCTCACCTCT

For transfection, the GenMute Reagent (Catalogue #SL100568, SignaGen, USA) was used. Normal microglia, astrocytes and stem cells (Hs27) were inoculated in groups of 300,000 cells in T25-flasks in 10% FBS at 37°C overnight. The next day, cells were starved in 1% FBS for 24 hrs. On the third day, the *CYP1B1* mutation was introduced into the target cells—that is the normal microglia, astrocytes and Hs27—separately via GenMute reagent using the CRISPR kit. The working solution of GenMute transfection was prepared by adding the CRISPR construct of the mutation. A second working solution was also prepared separately for the control, mock mutation (normal DNA sequence), which did not result in any amino acid changes in the target gene. All flasks were incubated for 4 hrs at 37°C after transfection. The media were then replaced with a fresh advanced DMEM-conditioned medium, which was the same medium used in the isolation step. The transfection efficiency of the reagent is around 99%, which was validated in our previous study [24]. However, damage to the DNA after transfection was not evaluated. Therefore, potential false positive results due to CRISPR activity should be considered when interpreting the results.

### Proliferation assay

Primary microglia and astrocytes obtained from rats were inoculated in groups of 5,000 cells per well in 96-well plates in 10% FBS at 37°C overnight. The next day, all cells were starved with DMEM (Catalogue #21885025, Thermo Fisher Scientific, USA) containing 1% FBS and were incubated overnight at 37°C. All cells were nourished with complete growth-specific media on the assay day. The *CYP1B1* gene was manipulated in both the microglia and the astrocytes. The control cells received mock manipulations. After 24 and 72 hrs, both the *CYP1B1*-edited microglia and astrocytes were compared with the control, mock-edited cells by measuring their metabolic activity using the MTT Cell Proliferation Assay (Catalogue #V13154, Thermo Fisher Scientific, USA). The company protocol was followed for assay performance. The signal was read via SpectraMax reader with SoftMax Pro v.6.2.1 software. Furthermore, similar conditions were applied to a commercial Hs27, human mesenchymal stem cell (fibroblast) line (Catalogue #CRL-1634, ATCC, USA), and then the MTT Assay was performed.

### Nicotinamide Adenine Dinucleotides Phosphates (NADP) reduction assay

For the assay, the amount of NADP and NADPH was determined in the biological samples. In the presence of NADP+ and NADPH, the enzyme reductase reduces a proluciferin reductase substrate to form luciferin (#G9081, Promega, USA). The total estimation of NADP/NADPH was reported. The measurement took place after 24 and 72 hrs of *CYP1B1* gene manipulation on rat microglia, astrocytes and Hs27 using the SpectraMax M5 reader and software (SoftMax Pro v.7.0.2).

### Phospho-kinase array

After 24 hrs of *CYP1B1* gene manipulation or mock manipulation in both microglia and astrocytes, cell lysates were collected from *CYP1B1*-edited microglia and astrocytes in addition to the collection of normal control lysate. All the lysates were prepared using RIPA solution (Catalogue #89901, Thermo Fisher Scientific, USA), which contains protease and phosphatase-inhibiting mini tablets (Product #88669, Thermo Fisher Scientific, USA). Protein quantitation was measured using a Microplate BCA Protein Assay Kit (Product #23252, Thermo Fisher Scientific, USA). A total of 300 μg of protein was used for each array membrane. The phospho-kinase array buffers were prepared and mixed with samples according to the manufacturer instructions (Catalogue #ARY003B, R&D system, USA). Signals were detected with chemiluminescent substrate (Product #34077, Thermo Fisher Scientific, USA) and were read using a ChemiDoc MP Imaging System and associated software (Image Lab v.5.1, S1 Fig). The analysis and estimation of proteins were both conducted using the Image J Program (v.1.5.2a). The graph bars were made using GraphPad PRISM v8.

### Bio-plex panel for inflammatory cytokines assay

The culture media of the *CYP1B1*-edited and control (mock) cells were collected for both astrocytes and microglia between the fifth and eighth days after manipulation, when the cells were confluent. The collected fractions of media were frozen at -80°C until use. The protocol of the Bio-Plex Pro Inflammation Panel 1 Assay was followed according to the manufacturer instructions (Catalogue #171-AL001M, Bio-Rad, USA). The samples were mixed separately with the kit’s beads in 96-well/ELISA plates. Each sample was inoculated in duplicates. Detection antibodies and streptavidin-PE were added as instructed with proper washing. The lower and upper limits of quantitation (LLOQ and ULOQ) were imputed from the standard curves included in the kit. The plate was covered with aluminium foil until it was installed in the Bio-Plex three-dimensional (3D) Suspension Array System (xPONENT software v.4.2.1441.0). The samples tested were conditioned with media collected from mutated microglia, control microglia, mutated astrocytes and control astrocytes. Conditioned media were collected from two independent experiments, and each sample was plotted in two different wells.

### Protein extraction and immunoblotting (western blot)

A minimum of 500,000 cells were used per assay. Similar to previously published protocols [24], the cells from *CYP1B1*-edited microglia and astrocytes were lysed, and the proteins were quantified. Likewise, the mock-edited microglia and astrocytes were lysed, and the proteins were quantified using a Microplate BCA Protein Assay Kit. Immunoblot analysis was performed by loading 20μg of protein samples on an SDS page of 10–20% gels (Life Technologies). Transfers were performed on wet transfer cells (Bio-Rad) with PVDF membranes (EMD Millipore). The antibodies used were anti-β-actin, internal control (Catalogue #3700, Cell Signaling Technology, USA), RXRα (Catalogue #3085, Cell Signaling Technology, USA), RXRβ (Catalogue #8715, Cell Signaling Technology, USA), RXRƳ (Catalogue #5629, Cell Signaling Technology, USA) and melatonin (*MTNR1B*) (Catalogue #ab203346, Abcam, USA). The secondary antibodies were goat anti-mouse (Catalogue #926–32210, Li-Cor, USA) and goat anti-rabbit (Catalogue #926–68021, Li-Cor, USA).

### Acquisition of Florescence Activated Cell Sorting (FACS) techniques

As previously described [25], samples containing live dissociated cells were stained for dead cell apoptosis using the annexin V Alexa Fluor antibody and propidium iodide kit (ThermoFisher #V13241) and were then analysed with the FACS CANTO II (BD Biosciences) Fluorescence-Activated Cell-Sorting Machine. Twenty-four hours before staining, the cells were manipulated with a mock mutation or the *CYP1B1* mutation (p.G61E). The background was deducted and defined with unstained cells.

### Statistical analysis

All experiments have been repeated three independent times, except for the phospho-kinase assay, which was performed twice. An unpaired t-test was used as the default for all comparisons. Each assay was normalised, and a simple comparison was made between the control and the test for each reading. An asterisk (*) was given when the p-value was less than or equal to 0.05.

## Results

### Decreased proliferation detection on *CYP1B1* mutated cells

**Fig 1:**
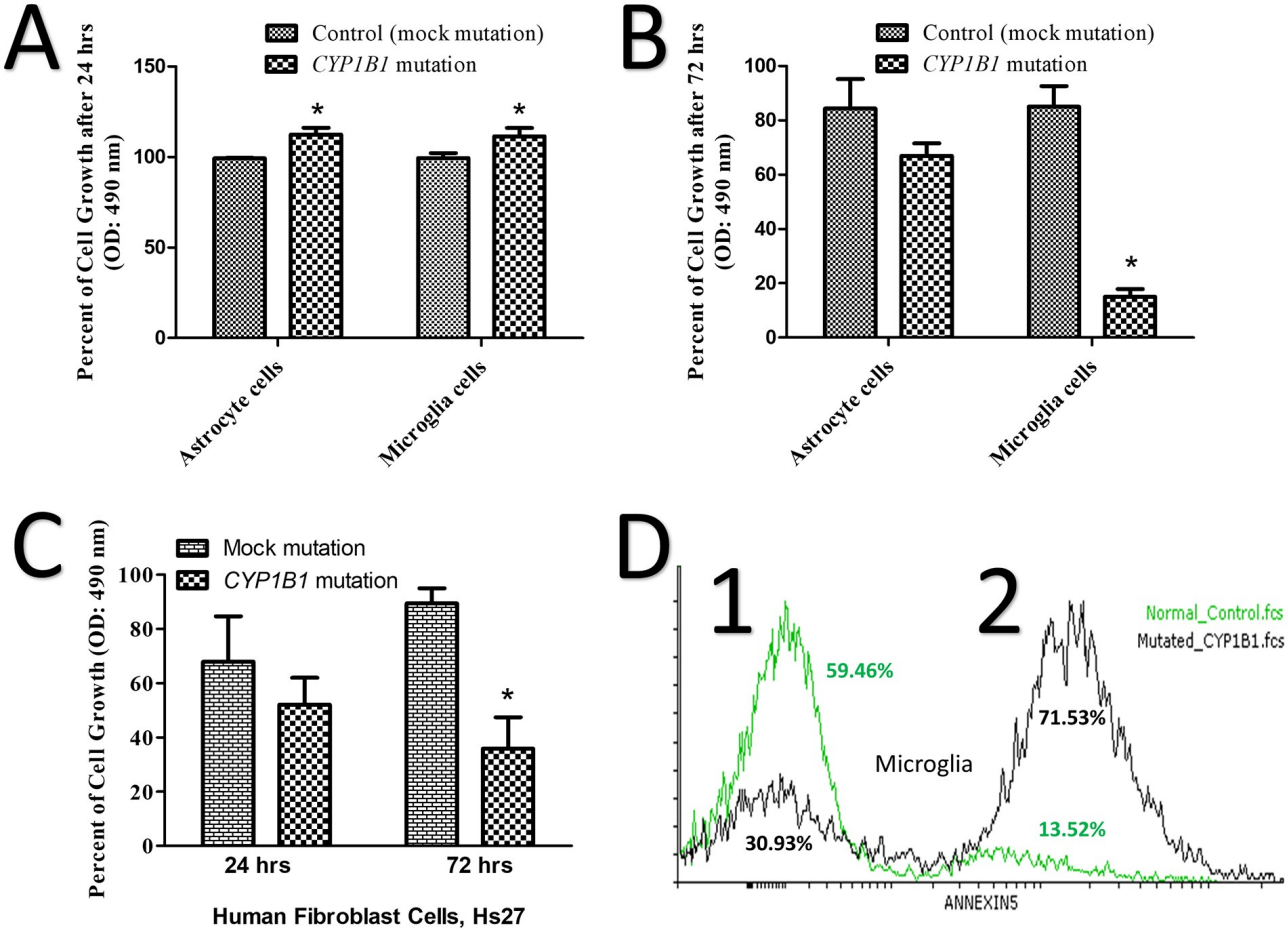
Mutant microglia show less cell growth and more apoptosis than the control. (A) A 10% increase in the growth of *CYP1B1* mutated astrocytes and microglia after 24 hrs. (B) After 72 hrs, growth was decreased in *CYP1B1* mutated microglia by 60%, but not in astrocytes. (C) The percentage of metabolic activities of the *CYP1B1* mutated mesenchymal stem cells line (Hs27) after 24 hrs and 72 hrs. (D) The assessment of microglia pre-apoptosis using an annexin V assay shows 71.53% pre-apoptotic cells in the mutated cells compared to 13.52% in the healthy control. An asterisk (*) is displayed when the p-value was less than or equal to 0.05; the number of experimental repeats (N) was three.

The mutated astrocytes and microglia, showed hyperactivity and an increase in proliferation of more than 10% within 24 hrs after introducing the CRISPR-edited *CYP1B1* (c.182G>A, p.Gly61Glu), compared to the control (Fig 1A; p < 0.05). Two days later, the microglia proliferation activity reduced by 60% in the CRISPR-mutated cells, compared to the control (Fig 1B; p < 0.05). The differences between the astrocytes and the control were not statistically significant (p = 0.214). In addition, the assessment of the human fibroblast (mesenchymal stem cells, Hs27) metabolic activities revealed no significant changes in the activity of the *CYP1B1*-mutated cells, in contrast to the control, within 24 hrs (Fig 1C; p = 0.482). After 72 hrs, the reduction in proliferation (metabolic) activities reached 40%, compared to the control, as shown in Fig 1C (p < 0.05).

### Changes in NADP/NADPH expression detected in *CYP1B1* mutated cells

**Fig 2:**
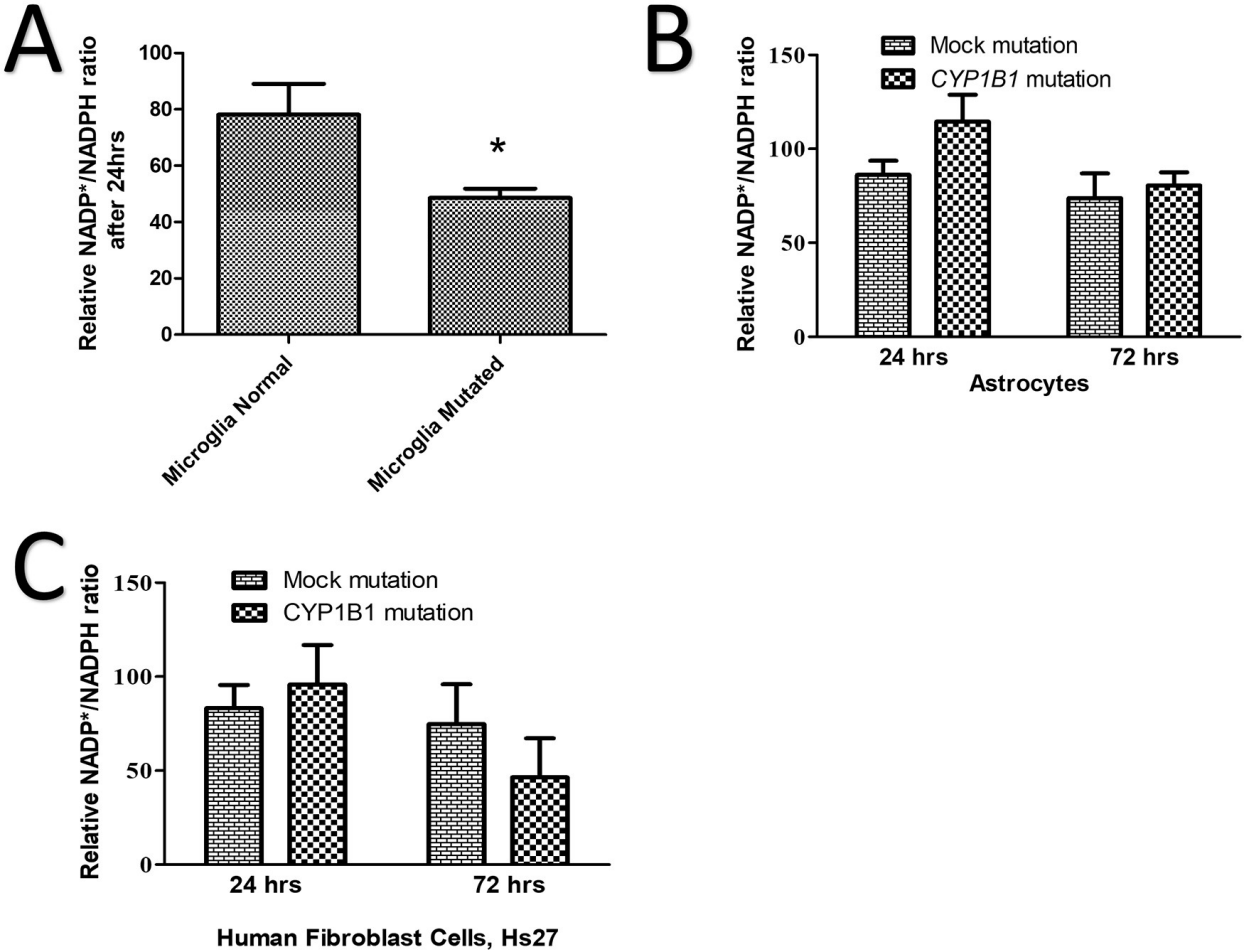
Mutated microglia show less reducing equivalent ratios than the control. (A) The normalised percentage of reducing equivalent ratio NADP*/NADPH decreased by 30% in mutant microglia compared to the normal control after 24 hrs. (B) The normalised percentage of the reducing equivalent ratio of NADP*/NADPH comparing the control (mock) astrocytes to mutated ones after 24 and 72 hrs, showing no significant difference. (C) The normalised percentage of the reducing equivalent ratio of NADP*/NADPH comparing the normal Hs27 to the mutated ones after 24 and 72 hrs, showing no significant difference. An asterisk (*) is displayed when the p-value was less than or equal to 0.05; N = 3.

The mutated microglia exhibited a significant 30% decrease (p < 0.05) in NADP/NADPH activity compared to the control (Fig 2A). In addition, the mutated *CYP1B1* genes in both the rat astrocytes and the human mesenchymal stem cells (Hs27), revealed no statistically significant changes in NADP/NADPH activity within 24 hrs, compared to the control (Fig 2B and 2C; p = 0.310). Similarly, the NADP/NADPH activity did not change after 72 hrs for either the mutated astrocytes or the Hs27, compared to the control (Fig 2B, p = 0.6761 and 2C, p = 0.392).

### Death of mutated microglia over time

We observed a tendency in the mutated microglia to die after three or more days in culture. Changing the media every day, prevented premature death due to the toxicity of too much debris. The flow cytometry analysis with annexin V (pre-apoptosis detection), which was conducted 24 hrs after *CYP1B1* or mock manipulation, showed 71% pre-apoptosis, compared to 13% in the control cells (Fig 1D).

### Deferential expression of phosphorylated proteins

**Fig 3:**
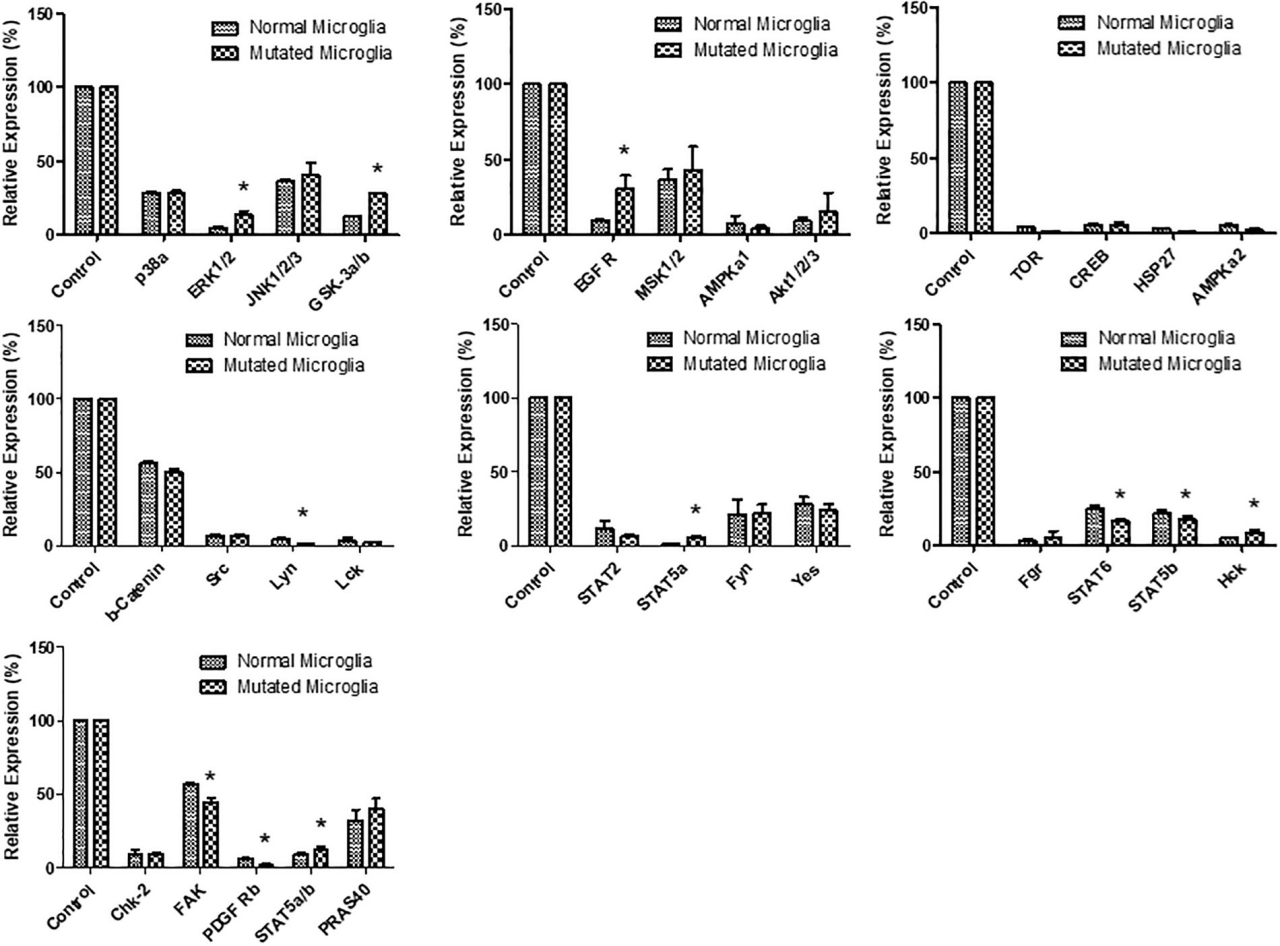
Normalised phospho-kinase expression of mutant microglia compared to a normal control. An asterisk (*) is displayed when the p-value was less than or equal to 0.05; N = 2.

**Fig 4:**
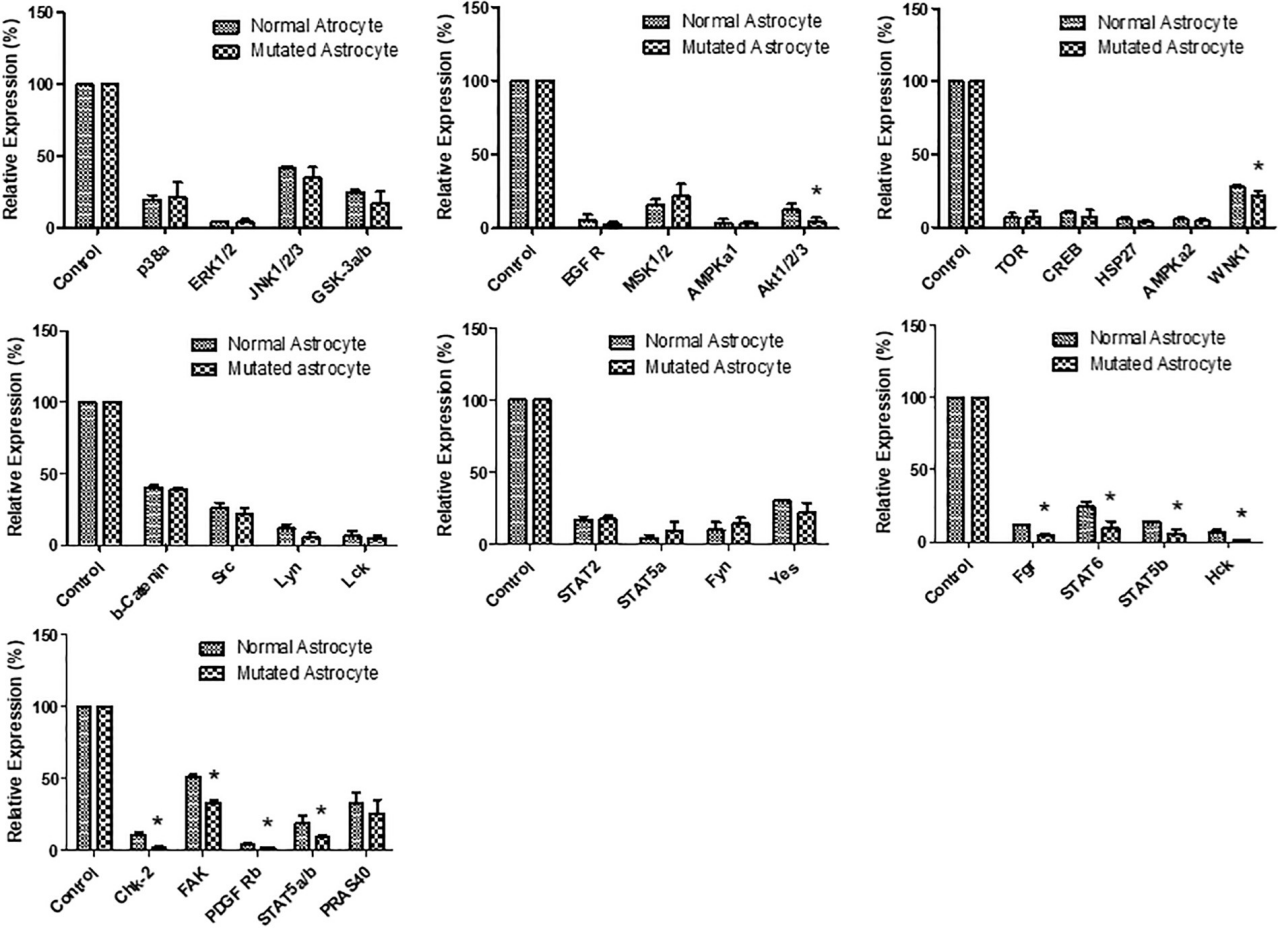
Normalised phospho-kinase expression of mutant astrocytes compared to a normal control. An asterisk (*) is displayed when the p-value was less than or equal to 0.05; N = 2.

The mutated microglia and astrocytes, showed differentially expressed phosphokinases, such as EGFR, ERK 1/2, GSK3 a/b, STAT3 and STAT5, compared to the control (See Figs 3 and 4). All of the above proteins are involved in proliferation-stimulating pathways [26]. The mutated microglia significantly (p < 0.05) expressed 11 of the 29 proteins tested, in comparison to the control cells (Fig 3 and S1 Table). In addition, the mutated astrocytes significantly (p < 0.05) expressed 10 of the 29 proteins tested, in comparison to the control (Fig 4 and S1 Table).

### Inflammatory cytokines detected in microglia and astrocytes

The mutated microglia and astrocytes, showed a total of 27 differentially expressed cytokines. Thirteen cytokines were significant (p < 0.05) between the mutated astrocytes and their control, whereas the remaining 14 cytokines were significant (p < 0.05) between the mutated microglia and their control (Table 1).

**Table 1:** Evaluation of cytokine expression in normal controls and in *CYP1B1* mutated cells.

	Cytokine Name	Expression in Normal Astrocytes N = 3	Expression in Mutated Astrocytes N = 3	Expression in Normal Microglia N = 3	Expression in Mutated Microglia N = 3
**1**	APRIL/TNFSF13 (42)	High	Low*	Not significant	Not significant
**2**	BAFF/TNFSF13B (37)	#	#	High	Low*
**3**	sCD30/TNFRSF8 (53)	Not detected	Not detected	#	#
**4**	sCD 163 (46)	Low	High*	#	#
**5**	Chitinase3-like1 (72)	High	Low#	Not detected	Not detected
**6**	gp130/sIL-6Rß (14)	High	Not detected	Not detected	High
**7**	IFN-a2 (20)	Not detected	High*	High	Low
**8**	IFN-ß (44)	#	#	Not significant	Not significant
**9**	IFN-g (21)	High	Low*	High	Low*
**10**	IL-2 (38)	Not significant	Not significant	High	Low*
**11**	SIL-6Ra (19)	High	Low*	#	#
**12**	IL-8 (54)	Not detected	Not detected	High	Not detected*
**13**	IL-10 (56)	Low	High*	Not significant	Not significant
**14**	IL-11 (39)	High	Not detected*	Not detected	Not detected
**15**	IL-12 (p40) (28)	Not significant	Not significant	High	Low*
**16**	IL-19 (29)	Not significant	Not significant	Not significant	Not significant
**17**	IL-20 (30)	Not detected	Not detected	Not detected	High*
**18**	IL-26 (22)	Not significant	Not significant	Not significant	Not significant
**19**	IL-27 (p28) (13)	#	#	Low	High*
**20**	IL-28A/IFN-Y2 (66)	High	Not detected*	Not significant	Not significant
**21**	IL-29/IFN-Y1 (33)	Not detected	Not detected	High	Not detected*
**22**	IL-32 (35)	Not detected	High*	Not detected	Not detected
**23**	IL-34 (15)	Not significant	Not significant	High	Low*
**24**	LIGHT/TNFSF14 (51)	High	Low*	High	Not detected*
**25**	MMP-1 (43)	Not detected	Not detected	#	#
**26**	MMP-2 (26)	Not detected	High*	High	Not detected*
**27**	Osteopontin(OPN) (77)	Not detected	High*	#	#
**28**	Pentraxin-3 (48)	Not detected	Not detected	High	Not detected*
**29**	sTNF-R1 (73)	Not detected	Not detected	Low	High*
**30**	sTNF-R2 (67)	High	Not detected*	High	Not detected*
**31**	TSLP (52)	Not significant	Not significant	Low	High*

^‘^*^’^ indicates significant results (p < 0.05). ‘Not detected’ indicates that the probe did not work or a negative result was reported.

^‘#’^ indicates that at least two probes out of four did not work, which means that the results probably need confirmation.

All data were obtained based on fixed standards supplemented in the kit. ‘High’ and ‘low’ labels were used based on the same standards. For concentrations, see S2 Table.

### Response of retinoic acid receptor and melatonin to mutations

**Fig 5:**
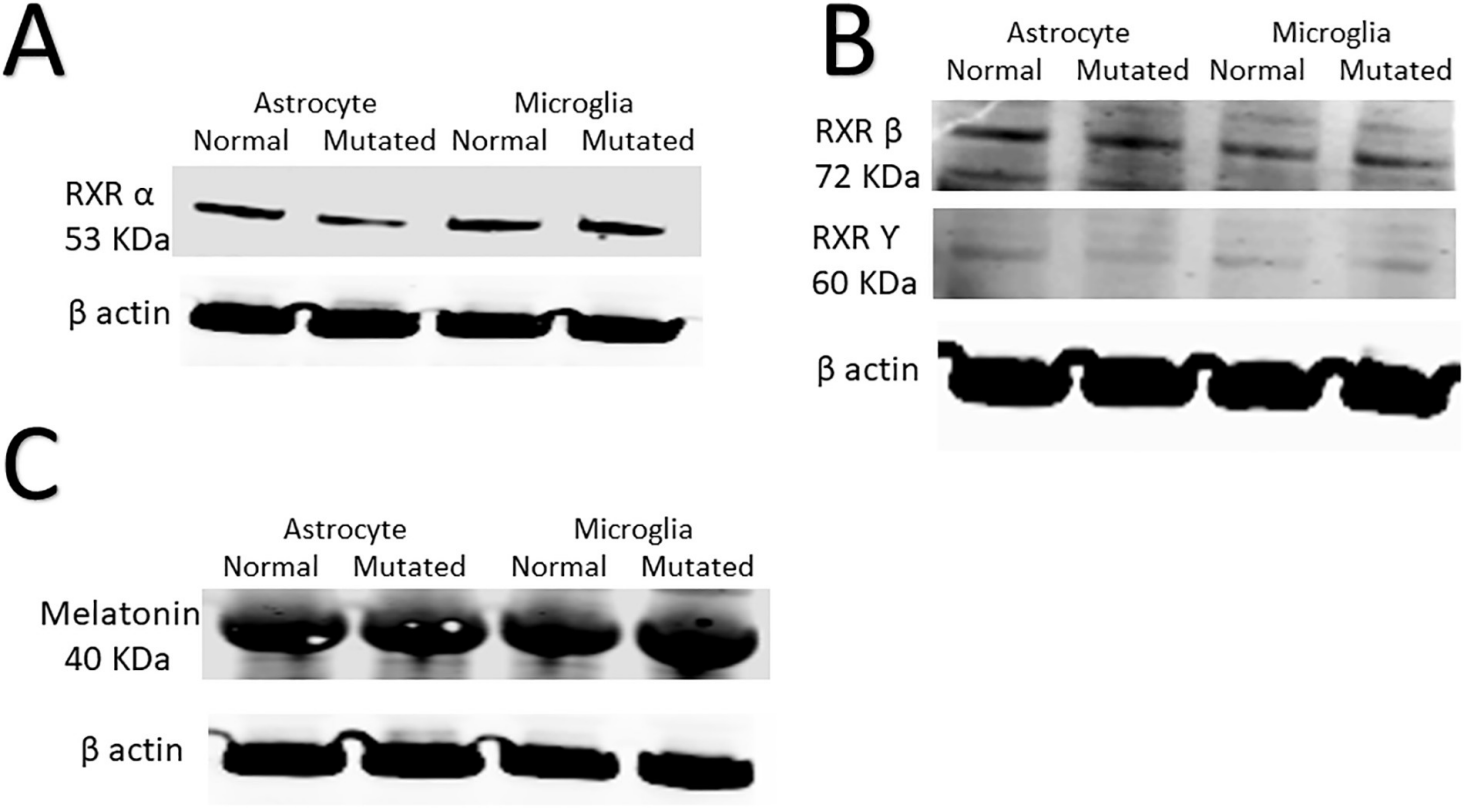
Immunoblots showing the expression of retinoic acid receptors (RXRs) and melatonin. (A) A western blot showing protein expression of RXR alpha (α) for mutant microglia and astrocytes compared to normal controls. (B) A western blot showing protein expression of RXR beta (β) and gFamma (Ƴ) in mutant microglia and astrocytes compared to normal controls. It is worth noting that RXRβ and RXRƳ were examined in the same blots. Therefore, the loading controls were reused for both entities. (C) A western blot showing the protein expression of melatonin for mutant microglia and astrocytes compared to normal controls; N = 3.

We observed a tendency in the mutated cells to decrease retinoic acid receptor (RXR) alpha (α), beta (β), and gamma (Ƴ). However, none of them exhibited a significant decrease in RXRα (p = 0.98), RXRβ (p = 0.89), or RXRƳ (p = 0.95) (Fig 5A and 5B). Melatonin was also non-significantly increased in the mutant microglia and astrocytes (Fig 5C).

## Discussion

The *CYP1B1* gene is one of the major genes mutated in PCG. Abu-Amero et al. have demonstrated a reduced probability for *CYP1B1* mutation to cause primary open-angle glaucoma in heterozygous patients [27]. However, more convincing evidence for the mutated gene’s contribution to primary congenital glaucoma PCG has been reported by Teixeira et al. which documented trabecular meshwork abnormalities in *CYP1B1* knockout mice (null mice) [28]. In addition, Safari et al. reported that the pG61E (p.Gly61Glu) mutation in the *CYP1B1* gene affects the extracellular matrix (ECM) in both humans and mice. ECM deficiency was documented in the human skin biopsies of two patients with a *CYP1B1* mutation [29]. The study suggested that the ECM damage was due to oxidative stress [30]. It is known that ECM is produced and repaired by mesenchymal stem cells [31]. Likewise, mesenchymal stem cells secrete anti-inflammatory mediators to protect retinal ganglion cells [32].

Our study investigated the effects of *CYP1B1* mutation (p.Gly61Glu) on the primary astrocytes and microglia of rats in addition to human skin mesenchymal stem cells. We acknowledge that astrocytes and microglia isolated from rat brains may not be the same as cells isolated from the optic nerve head of rats. This limitation may contribute to invalid conclusions. Mutated astrocytes and microglia showed a 10% increase in proliferation in the first 24 hrs, (Fig 1A), which is most likely due to the activation of both cells. This increase, however, was not long lasting, and the increase in activity dropped by an average of 40% in 72 hrs (Fig 1B), suggesting that the activation was temporary. The decrease in proliferation at 72 hrs was statistically significant in microglia only (Fig 1B), suggesting that the *CYP1B1* mutation effect was more severe in the microglia. We should account for interference of the terms proliferation, cell growth and hyperactivity when interpreting metabolic activity data obtained at 24 hrs or more. Investigating oxidative stress in astrocytes and microglia revealed a reduction in the NADP/NADPH ratio in microglia only (Fig 2A and 2B). The reducing equivalent in the microglia which was declined by 20–45%, may contribute to the pathogenesis of reactive oxygen species (ROS). Skin mesenchymal stem cells (Hs27) play a major role in ECM repair and ROS inhibition [33]. Hs27 were tested under the stress of the p.Gly61Glu mutation in the *CYP1B1* gene and showed a 40% reduction in their ability to proliferate after 72 hrs (Fig 1C). This suggests potentially less repair to the ECM or ROS inhibition when damage to the environment occurs. Furthermore, it suggests less protection to retinal ganglion cells when progressive inflammation is present. Luckily, evaluating the NADP/NADPH ratio within *CYP1B1*-mutated Hs27 revealed no significant ROS abnormality compared to the control (Fig 2C). With the above results, we have supported the findings of other studies by confirming the existence of a potential leak of free radicals into the microenvironment. Additionally, the mutated microglia showed 71% pre-apoptosis, suggesting programmed cell death within a few days (Fig 1D). High numbers of dead and activated microglia contribute to elevated inflammation. However, we suggest that microglia probably contribute to the ROS leak in PCG, among other factors.

We report upregulation of ERK1/2, GSK3a/b, EGFR, STAT5a and Hck cytokines in mutant microglia (Fig 3). It was previously discovered that ERK1/2 and EGFR promote a pro-inflammatory response [34]. In addition, GSK3a/b is a key protein known to regulate the balance between pro-inflammatory and anti-inflammatory responses [35]. Microglia proliferation is reduced when either STAT5 or HcK is inhibited [36]. The Hck gene is known to be upregulated in activated microglia [37]. Furthermore, we report down-regulation of Lyn, STAT6, STAT5b, focal adhesion kinase (FAK) and PDGF-Rb cytokines in mutant microglia. Microglia migration is controlled by Lyn [38]. In microglia, the clearance of dead cells, debris and the resolution of inflammation are promoted by STAT6. Similarly, cell adhesion, proliferation, migration, survival and pro-inflammation are mediated by FAK in microglia [39]. Microglia expressing PDGFRB have been found to promote vascular wall proliferation and repair [40].

In our study, the mutant astrocytes down-regulated the following phosphorylation proteins: akt1/2/3, WNK1, Fgr, STAT6, STAT5b, Hck, Chk2, FAK, PDGF Rb and STAT5a/b (Fig 4). The reduction of WNK1 is known to attenuate neural pain and reactive astrocytosis through NKCC1 inhibition [41]. Neural apoptosis is reportedly mediated by Hck [42]. Activation of akt1/2/3 and STAT6 in astrocytes promotes an anti-inflammatory response, as found in a previous study [43]. The damage response of DNA increases Chk2 expression in astrocytes, as found in a previous study [44].

In mutant astrocytes, we report upregulation of the following cytokines compared to the control: sCD63, IFN-a2, IL-10, IL-32, MMP-2 and osteopontin (Table 1 and S2 Table). Out of these cytokines, IFN-a2, IL-32, sCD63 and osteopontin are pro-inflammatory, whereas IL-10 is the only anti-inflammatory cytokine upregulated [45]. IL-10’s status as an anti-inflammatory cytokine suggests activation of the feedback loop that is activated when inflammation progresses for a long period of time [46]. In addition, mutant astrocytes over-produce MMP-2, which is involved in ECM breakdown. This over-production probably increases the severity of ECM breakdown under inflammatory conditions.

In mutant microglia, we report upregulation of the following cytokines compared to the control: gp130, IL-20, IL-27 (p28) and TSLP (Table 1 and S2 Table). These cytokines are involved in microglia activation and neural protection [47]. *CYP1B1* and retinoic acid receptors (RXR) contribute to the maintenance of the homeostasis of the following endogenous complexes: steroid hormones, fatty acids, melatonin and vitamins [13]. In addition, *CYP1B1* has been reported to be important for nervous system and vision health by interacting with and regulating melatonin metabolism. Melatonin is produced in the retina, lacrimal gland, lens and ciliary body of the eye [48]. On the other hand, melatonin has been reported to inhibit *CYP1B1* via a feedback loop [13]. The expression of RXR isoforms and melatonin (*MTNR1B*) in the context of astrocytes and microglia has been investigated. Decreased *CYP1B1* expression reduces fatty-acid oxidation, which involves RXR activities [13]. We report that RXRα, RXRβ and RXRƳ did not change in either mutated astrocytes or mutated microglia (Fig 5A and 5B). It seems that RXR isoforms did not respond to *CYP1B1* inhibition. In addition, no significant changes in melatonin were observed after mutating the *CYP1B1* gene (Fig 5C). This result was interesting because others have reported a link between *CYP1B1* expression and melatonin expression [49].

Melatonin was previously reported to stimulate neuronal cell survival involving the Akt/NF-κB pathway [50]. Most likely, melatonin homeostasis within astrocytes and microglia differs from the current understanding in the published research.

Based on the aforementioned data, we conclude that the p.Gly61Glu mutation within the *CYP1B1* gene induces microglial response. Additionally, the mutation reduced proliferation in all tested cell types. Furthermore, the mutation tended to increase ROS and apoptosis, which contribute to damaging effects on the ECM and/or trabecular meshwork. Likewise, the increased production of MMp-2 further damages the ECM in the environment or tissue. Moreover, microglia are important for retinal ganglion cell survival because they play an important role in clearing pathogens and inflammation [51]. Severe *CYP1B1* mutation may damage eye development through loss of microglia, decreased ECM and increased ROS. We suggest more experimental testing to understand whether the identified progressive inflammation is preventable via non-invasive procedures, such as drugs. We also suggest investigating whether progressive inflammation is a cause for failure of glaucoma trabeculectomy, which is a surgery to remove eye-drainage tubes to allow fluid to drain smoothly.

## Supporting information

S1 Table Normalised raw data for phospho-kinase expression of astrocytes and microglia before and after *CYP1B1* manipulation. (DOCX)

S2 Table Cytokine concentrations (pg/ml) evaluated in normal controls and *CYP1B1* mutated cells, which show differential differences. (DOCX)

S1 Fig Blots resulting from a phospho-kinase assay showing reactivity in mutated astrocytes, normal astrocytes, mutated microglia and normal microglia. (TIF)

S2 Fig (A) shows isolated astrocytes stained with GFAP (red) and the nucleus stained with DAPI (blue). Positive cells stained with GFAP represent 93.9% of total cells. (B) shows isolated microglia stained with CD64 (green) and the nucleus stained with DAPI (blue). Positive cells stained with CD64 represent 91% of the total cells. In addition, there was no cross-contamination with astrocytes in the isolated microglia (B); no positive GFAP cells were found.
(TIF)

S1 Raw image (TIF)
