# Supplementary material for: The loss of microglia activities facilitates glaucoma progression in association with CYP1B1 gene mutation (p.Gly61Glu)
Source: PLoS One. 2020 Nov 10;15(11):e0241902. doi: 10.1371/journal.pone.0241902 (PMC7654781; doi:10.1371/journal.pone.0241902)
Supplement: S1 Table — (DOCX) [file pone.0241902.s001.docx]

S1 Table. normalized raw data of significant Phospho-kinase expression of astrocytes and microglia before and after *CYP1B1* manipulation.

| **No.** | **Name** | **Normal Astrocytes** | | **Mutated Astrocytes** | | **P value** |
| --- | --- | --- | --- | --- | --- | --- |
| 1 | Akt1/2/3 | 8.304361 | 16.507740 | 2.151552 | 6.932095 | 0.0138 |
| 2 | WNK1 | 26.412240 | 29.276060 | 24.995040 | 18.791990 | 0.051 |
| 3 | Fgr | 11.235730 | 11.871640 | 5.780475 | 3.501143 | 0.0281 |
| 4 | STAT6 | 27.262860 | 20.946220 | 14.389660 | 4.239631 | 0.050 |
| 5 | STAT5b | 12.665680 | 14.430360 | 2.988373 | 8.942981 | 0.0470 |
| 6 | Hck | 8.454602 | 4.255212 | 0.4999643 | 1.376857 | 0.0259 |
| 7 | Chk-2 | 8.900524 | 12.687300 | 1.940832 | 3.215281 | 0.0500 |
| 8 | FAK | 49.598180 | 52.769220 | 34.977100 | 31.165210 | 0.0182 |
| 9 | PDGF Rb | 4.583866 | 4.876077 | 2.356936 | 0.9533372 | 0.050 |
| 10 | STAT5a/b | 13.616400 | 24.339680 | 8.323088 | 10.585470 | 0.0500 |
| 11 | p53 | 26.90080 | 23.89354 | 0.00000 | 0.00000 | 0.0035 |

| **No.** | **Name** | **Normal Microglia** | | **Mutated Microglia** | | **P value** |
| --- | --- | --- | --- | --- | --- | --- |
| 1 | ERK1/2 | 5.325852 | 3.956620 | 10.210160 | 16.119360 | 0.0500 |
| 2 | GSK-3a/b | 12.433090 | 11.994260 | 28.617120 | 27.027180 | 0.0028 |
| 3 | EGF R | 8.952021 | 9.713581 | 21.797260 | 39.168530 | 0.0178 |
| 4 | Lyn | 5.265999 | 2.913895 | 0.9304468 | 0.9609585 | 0.0214 |
| 5 | STAT5a | 1.282587 | 1.076267 | 6.211436 | 5.303813 | 0.0102 |
| 6 | STAT6 | 27.260810 | 21.607480 | 17.768670 | 14.876290 | 0.0500 |
| 7 | STAT5b | 24.063790 | 18.690580 | 15.297950 | 19.392050 | 0.3547 |
| 8 | Hck | 5.302189 | 5.155126 | 6.668256 | 10.873830 | 0.0499 |
| 9 | FAK | 55.922000 | 57.742920 | 46.775760 | 42.471810 | 0.011 |
| 10 | PDGF Rb | 6.058774 | 6.628439 | 2.790430 | 1.589522 | 0.0217 |
| 11 | STAT5a/b | 7.885235 | 9.852256 | 10.374250 | 14.598610 | 0.0713 |
