# Supplementary material for: The loss of microglia activities facilitates glaucoma progression in association with CYP1B1 gene mutation (p.Gly61Glu)
Source: PLoS One. 2020 Nov 10;15(11):e0241902. doi: 10.1371/journal.pone.0241902 (PMC7654781; doi:10.1371/journal.pone.0241902)
Supplement: S2 Table — (DOCX) [file pone.0241902.s002.docx]

S2 Table. Cytokines concentration (pg/ml) evaluated in normal control and *CYP1B1* mutated cells.

|  | Cytokine Name | Normal Astrocyte  N=3  (pg/ml) | Mutated Astrocyte  N=3  (pg/ml) | Normal Microglia  N=3  (pg/ml) | Mutated Microglia  N=3  (pg/ml) |
| --- | --- | --- | --- | --- | --- |
| 1 | APRIL/TNFSF13 (42) | 15200 ± 2000 | 4200 ± 3600* | 13950 ± 900 | 16450 ± 5100 |
| 2 | BAFF/TNFSF13B (37) | 375 ± 150 | 700 ± 700 | 3075 ± 350 | 1450 ± 700* |
| 3 | sCD30/TNFRSF8 (53) | Not detected | Not detected | 5.2 ± 2.6 | 1.4 ± 0.7 |
| 4 | sCD 163 (46) | 1781 ± 2350 | 5767.5 ± 1445* | 1950 ± 225 | 1700 |
| 5 | Chitinase3-like1 (72) | 75 ± 37 | 55 ± 27 | Not detected | Not detected |
| 6 | gp130/sIL-6Rß (14) | 365 ± 182.5* | Not detected | Not detected | 380 ± 190* |
| 7 | IFN-a2 (20) | Not detected | 90 ± 60* | 210 ± 40 | 60 ± 60 |
| 8 | IFN-ß (44) | 305 ± 130 | 275 ± 30 | 317.5 ± 53 | 165 ± 148 |
| 9 | IFN-g (21) | 592.5 ± 135 | 275 ± 50* | 475± 70 | 265 ± 50* |
| 10 | IL-2 (38) | 40 ± 20 | 80 ± 60 | 205 ± 30 | 60 ± 60* |
| 11 | SIL-6Ra (19) | 150.8 ± 28.5 | 52.5 ± 31* | 93 ± 84 | 106.5 ± 97 |
| 12 | IL-8 (54) | Not detected | Not detected | 320 ± 220 | Not detected* |
| 13 | IL-10 (56) | 36.88 ± 15.25 | 83.13 ± 13.7* | 26.5 ± 23 | 19.5 ± 1 |
| 14 | IL-11 (39) | 187.5 ± 55 | Not detected* | Not detected | Not detected |
| 15 | IL-12 (p40) (28) | 490 ± 140 | 425 ± 150 | 535 ± 70 | 342.5 ± 45* |
| 16 | IL-19 (29) | 860 ± 300 | 615 ± 130 | 925 ± 459 | 1005 ± 431 |
| 17 | IL-20 (30) | Not detected | Not detected | Not detected | 38 ± 26* |
| 18 | IL-26 (22) | 180 ± 60 | 175 ± 130 | 492.5 ± 65 | 370 ± 60 |
| 19 | IL-27 (p28) (13) | 950 ± 1345 | 900 ± 1131 | 1175 ± 265 | 200 ± 141* |
| 20 | IL-28A/IFN-Y2 (66) | 250 ± 100 | Not detected | 620 ± 200 | 410 ± 100 |
| 21 | IL-29/IFN-Y1 (33) | Not detected | Not detected | 670 ± 500 | Not detected* |
| 22 | IL-32 (35) | Not detected | 5 ± 2* | Not detected | Not detected |
| 23 | IL-34 (15) | 1130 ± 40 | 1010 ± 200 | 1175 ± 600 | 670 ± 800 |
| 24 | LIGHT/TNFSF14 (51) | 190 ± 45 | 37 ± 15* | 39 ± 18 | Not detected* |
| 25 | MMP-1 (43) | Not detected | Not detected | 1040 ± 920 | 20± 18 |
| 26 | MMP-2 (26) | Not detected | 2000 ± 1000* | 8350 ± 5000 | Not detected* |
| 27 | MMP-3 (45) | 3700 ± 3000 | 6450 ± 1300 | 7525 ± 350 | 7250 ± 5100 |
| 28 | Osteopontin(OPN) (77) | Not detected | 1215 ± 670* | 1025 ± 650 | 500 ± 250 |
| 29 | Pentraxin-3 (48) | Not detected | Not detected | 32 ± 17 | Not detected |
| 30 | sTNF-R1 (73) | Not detected | Not detected | 105 | 320* |
| 31 | sTNF-R2 (67) | 290 ± 150 | Not detected* | 410 ± 200 | Not detected* |
| 32 | TSLP (52) | 10 ± 5 | 70 ± 35 | 66 ± 8 | 195 ± 22* |

(*) means significant result (p < 0.05). (Not detected) means the probe didn’t work or negative result was reported.
