# Supplementary figures and images for: The loss of microglia activities facilitates glaucoma progression in association with CYP1B1 gene mutation (p.Gly61Glu)
Source: PLoS One. 2020 Nov 10;15(11):e0241902. doi: 10.1371/journal.pone.0241902 (PMC7654781; doi:10.1371/journal.pone.0241902)

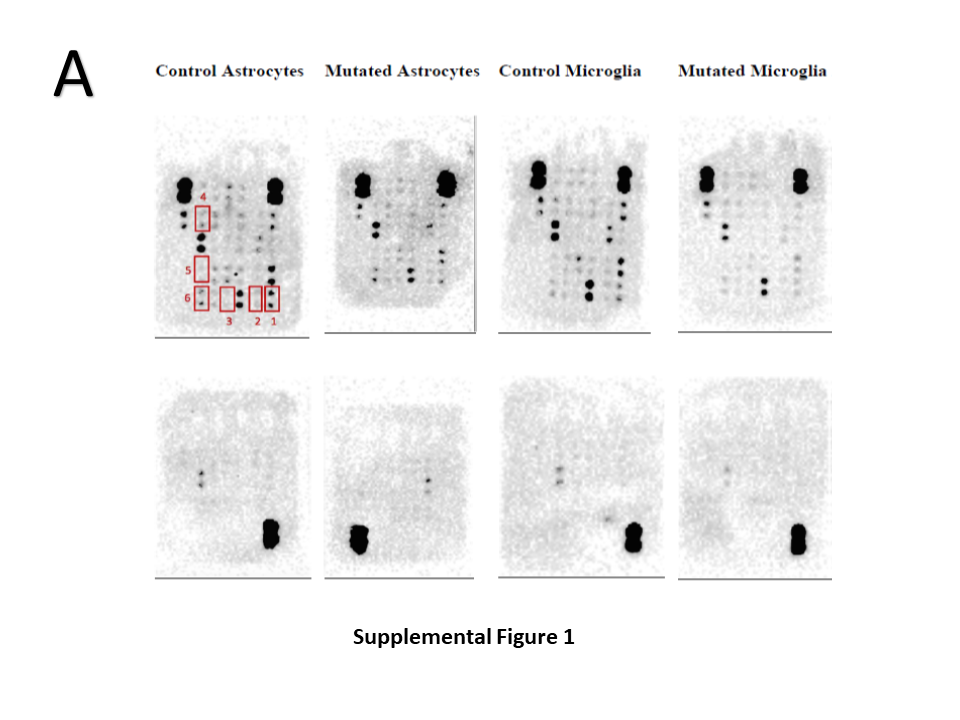

Supplement: S1 Fig — (TIF) [file pone.0241902.s003.tif]

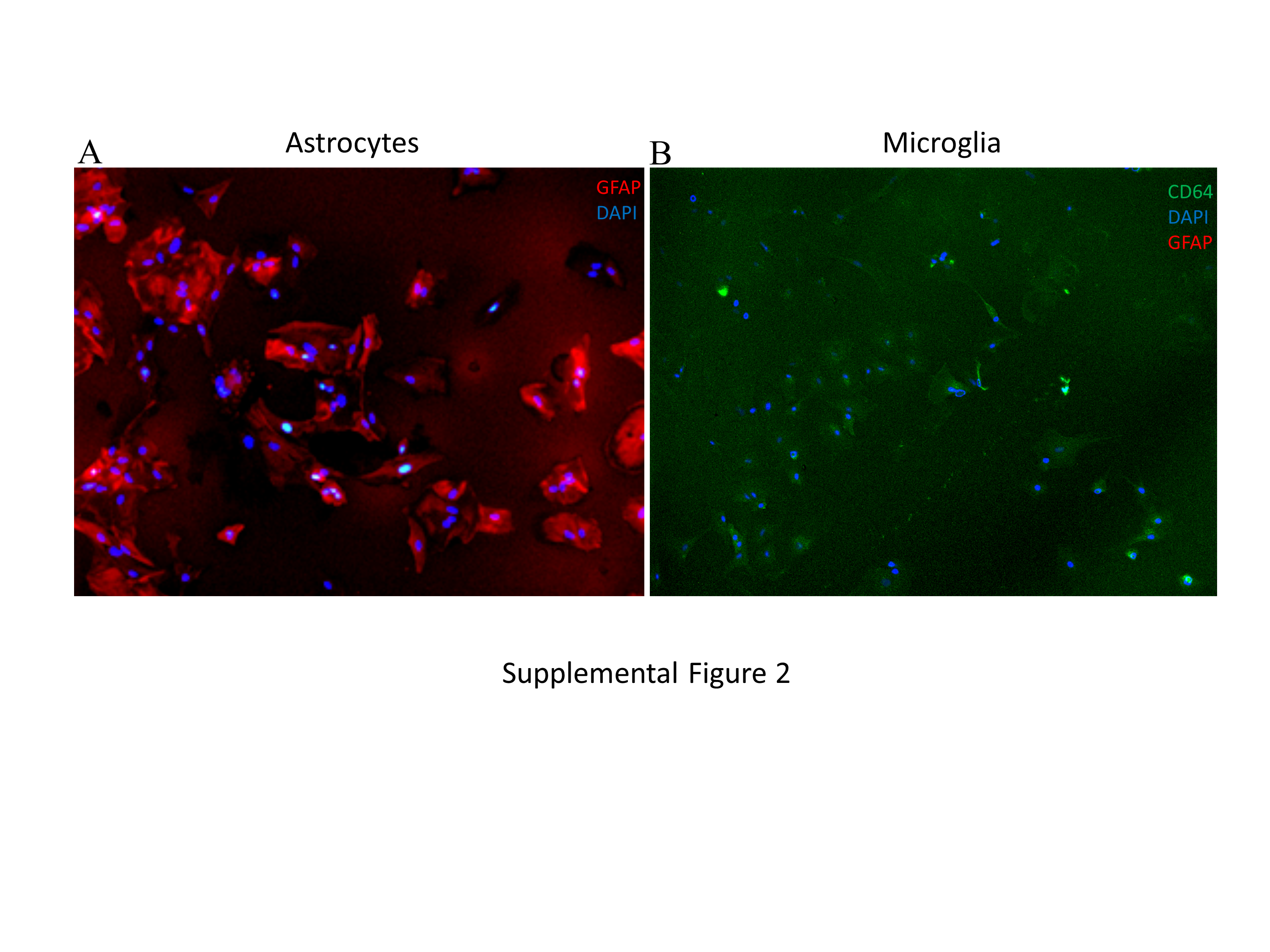

Supplement: S2 Fig — (A) shows isolated astrocytes stained with GFAP (red) and the nucleus stained with DAPI (blue). Positive cells stained with GFAP represent 93.9% of total cells. (B) shows isolated microglia stained with CD64 (green) and the nucleus stained with DAPI (blue). Positive cells stained with CD64 represent 91% of the total cells. In addition, there was no cross-contamination with astrocytes in the isolated microglia (B); no positive GFAP cells were found. (TIF) [file pone.0241902.s004.tif]

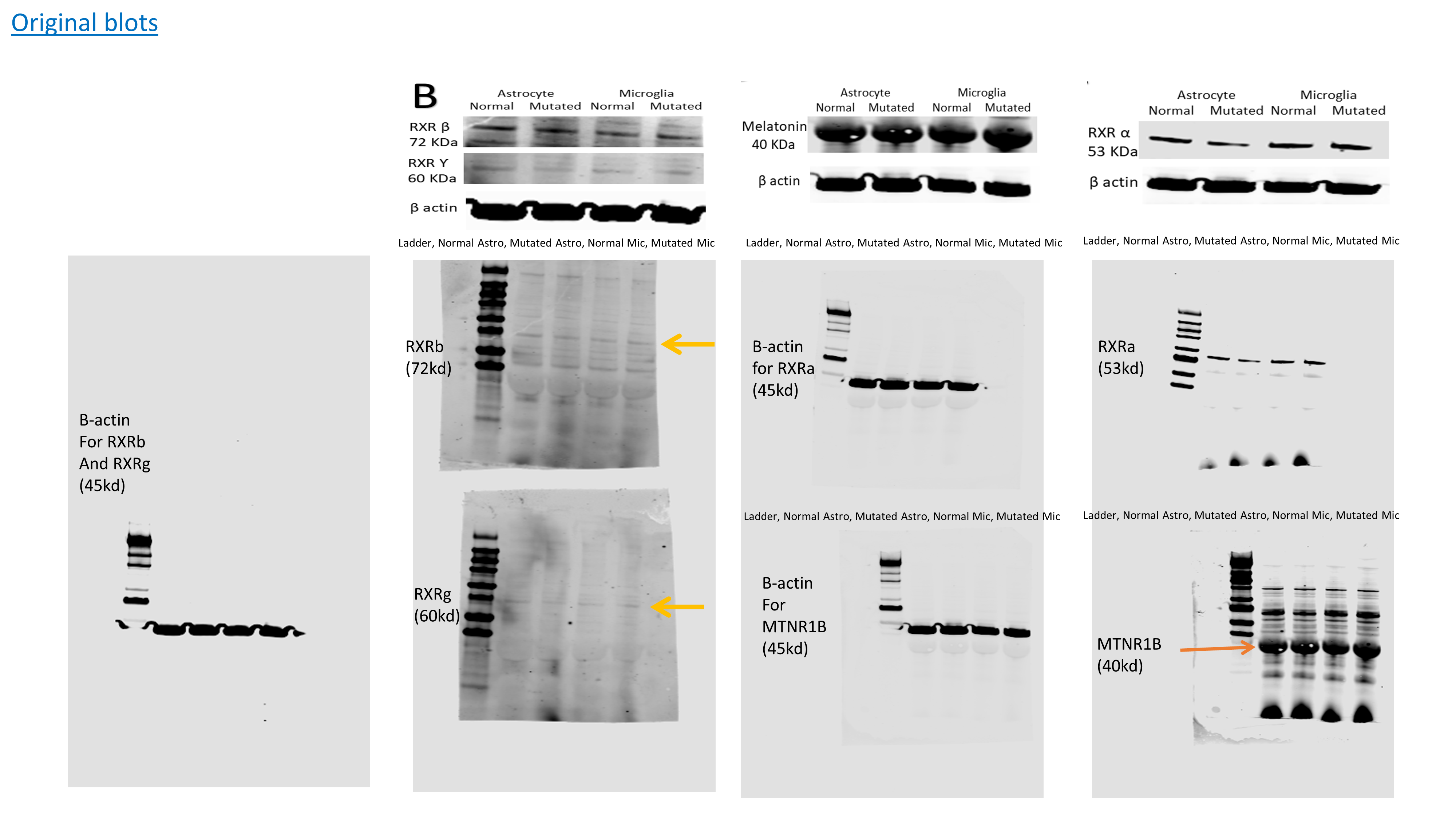

Supplement: S1 Raw image — (TIF) [file pone.0241902.s005.tif]
